# Developmental Plasticity and the Evolutionary Rescue of a Colonizing Mite

**DOI:** 10.1111/ede.70002

**Published:** 2025-02-18

**Authors:** Kathryn A. Stewart, Isabel M. Smallegange

**Affiliations:** ^1^ Institute of Environmental Sciences (CML) Leiden University Leiden the Netherlands; ^2^ School of Natural and Environmental Sciences Newcastle University Newcastle upon Tyne UK

**Keywords:** eco‐evolutionary dynamics, phoresy, polyphenism

## Abstract

Plasticity, especially in small newly founded populations, can expose genetic variation to selection during the evolutionary rescue of populations, allowing individuals to achieve a phenotype with which they can survive. However, developmental plasticity can also enable organisms to accommodate perturbations, generating new phenotypic variation. We explored whether, at the start of a colonization event, phenotype dynamics follow a “selective” process in which plasticity fuels evolutionary rescue or whether they are due to developmental plasticity in a “generative” process. We investigated this using the bulb mite *Rhizoglyphus robini*, which expresses a facultative, juvenile dispersal phenotype (deutonymph) under unfavorable conditions and shows alternative adult male phenotypes: competitive fighters or benign scramblers that are expressed to mitigate food stress and which have higher levels of genetic heterozygosity than fighters. Mimicking colonization dynamics, we founded small, medium and large populations from deutonymphs on low or high food to test if scramblers were expressed earliest postcolonization within (i) the *smallest* founder populations to alleviate inbreeding (selective hypothesis), or (ii) the *largest* founder populations as a direct consequence of food stress is highest due to higher food competition (generative hypothesis). In line with the generative hypothesis under both food environments, scramblers were expressed at the earliest in the largest founder populations, which also tended to show the lowest growth at the start of the experiment and had the lowest ultimate population size. Our findings highlight the necessity to seek explanations of how developmental pathways likely influence evolutionary rescue patterns, starting with how resource limitation (stress) shapes adaptive responses during colonization.

## Graphical abstract

The study investigates the role of plasticity in small populations during evolutionary rescue, using the bulb mite *Rhizoglyphus robini*. It explores whether phenotype dynamics of colonizing populations comprising only dispersers (deutonymphs) are due to a selective process (plasticity exposing genetic variation) or a generative process (developmental plasticity). The research found that scramblers, which, as opposed to energetically expensive fighters, are an alternative adult male phenotype indicating stress mitigation during development, were expressed earliest in the largest founder populations under two food environments, supporting the generative hypothesis. This work highlights the importance of understanding how developmental pathways and resource limitations shape adaptive responses during colonization.

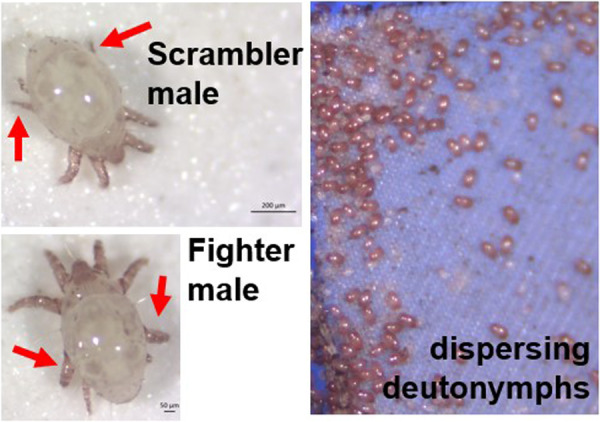

## 1 Introduction

Most organisms experience environmental heterogeneity or unpredictability due to various small‐ (e.g., flood, fire) and large‐scale (e.g., invasive species, climate change) factors that regulate critical resources. Organisms are inherently responsive to such changes and modify their morphology and physiology to fit their surroundings (Smallegange and Deere 2014), such as switching between food sources when nutrients become depleted (Moran 1994) or moving to new locations where conditions are more favorable (Bonte et al. 2012). However, establishing whether such short‐term environmental responsiveness, or plasticity, is related to long‐term evolutionary patterns is a persistent problem in biology (Lande 2015; Uller et al. 2024).

Arguments exist that plasticity plays an important role in the evolutionary rescue of populations (Chevin et al. 2013; Uller et al. 2024), as well as in the population establishment of novel environments (Lande 2009). For example, current evolutionary models posit that plasticity drives adaptive phenotypic change by exposing (cryptic) genetic variation to selection (Levis and Pfennig 2016; Dayan et al. 2019), fueling adaptive evolution (Buzatto et al. 2015). These models capture how plasticity impacts population‐level characteristics, including phenotypic distributions. But developmental processes often enable organisms to accommodate perturbations (West‐Eberhard 2003) so that the distribution of phenotypes in a population will also depend on how development operates (Salazar‐Ciudad 2021; Deere and Smallegange 2023). However, because the latter evolutionary models do not recognize developmental plasticity as a source of adaptive phenotypic variation, they cannot explain *why* and *how* particular phenotypes appear (Uller et al. 2024). As a result, they cannot disentangle adaptive change resulting from developmentally generated variation that selection can act upon (“generative” process) versus selection alone (“selective” process) (Uller et al. 2020; Schlichting 2021; Uller et al. 2024).

Invertebrates are an excellent taxonomic group to test to what extent “generative” or “selective” processes explain the evolutionary rescue of populations because many show high colonization rates (Bonte et al. 2012), high sensitivity to environmental change (Barnes et al. 2009) and high levels of plasticity (Brockmann 2008). For example, the bulb mite *Rhizoglyphus robini* (Acari: Acaridae) not only shows a facultative, juvenile dispersal phenotype, the deutonymph (Deere et al. 2015), which is expressed in response to deteriorating environmental conditions and is likely the primary initiator of new populations during colonization; it also has two alternative, adult male phenotypes: fighter males and scrambler males (Smallegange 2011, but see Stewart et al. 2018). Adult fighter males metamorphose under high resource availability to possess a thickened third leg pair with dagger‐like claws with which they can kill conspecifics (Smallegange 2011). Juvenile males under low resource conditions also metamorphose into an adult, however, they instead express the scrambler phenotype that does not have such leg modifications (Rhebergen et al. 2022). Heritability estimates for the scrambler and fighter morphs in our stock cultures are 0.41 and 0.30, respectively (Smallegange and Coulson 2011), suggesting that heritability of male morph results from polygenic condition‐dependence. Expression of fighters versus scramblers is often considered to be sexually selected (e.g., Łukasiewicz et al. 2020; Parrett et al. 2022), but empirical evidence also suggests it is strongly regulated by the developmental environment, namely the dynamics of population density, food competition, and individual energy economies (Smallegange et al. 2019; Rhebergen et al. 2022). For example, when mites have ad libitum access to high‐quality food (yeast), inbred lines derived from scrambler males have increased inbreeding depression compared to inbred lines derived from fighter males (Łukasiewicz et al. 2020; Parrett et al. 2022). Interestingly, however, in stressful environments, scramblers show higher levels of genetic heterozygosity (Stewart et al. 2019) and higher siring rates (Van den Beuken et al. 2019a) than fighters, potentially enabling (cryptic) genetic variation to facilitate population persistence. Inversely, bulb mite populations with high fighter expression are more prone to extinction under environmental change (Łukasiewicz et al. 2023). Finally, deutonymphs, which establish new founder populations, have only been observed to metamorphose and develop into adult females and fighters, albeit this phenomenon has only been tested under favorable environmental conditions (Smallegange and Coulson 2011; Deere et al. 2015).

The life history characteristics of the bulb mite allow us to test how developmental plasticity can impact phenotype dynamics and adaptive evolution following colonization: via the exposure of (cryptic) genetic variation, a “selective” process, or because organisms accommodate postcolonization conditions through developmental plasticity in a “generative” process. Specifically, if newly founded populations differ in size, all else being equal, our “selective” hypothesis states that scramblers should be expressed *earlier* in smaller than larger founder populations postcolonization to alleviate potential inbreeding depression and rescue populations (Stewart et al. 2019). However, if scrambler expression is a response to postcolonization conditions, our “generative” hypothesis states that, following colonization, scramblers should be expressed *later* in smaller than larger founder populations. In this scenario, we posit that per capita food availability will be higher in smaller than larger founder populations allowing fewer juvenile males to suffer food stress. This then allows juveniles to survive maturation when investing in costly fighter morphology early in the colonization process (as opposed to mitigating food stress by developing less‐costly scrambler morphology in larger founder populations) (Deere and Smallegange 2023). To try and differentiate these two mechanisms and their ensuing consequences to population establishment and persistence, we tested these predictions in an eco‐evolutionary experiment in which we founded small, medium, and large populations from deutonymphs. In each founder population, we evolved these mites in either a good or bad food environment, and then followed them for approximately 7–10 overlapping generations. Previous population experimental work (Smallegange 2011b; Smallegange and Deere 2014) demonstrated generation times to vary from egg‐to‐egg between 11 and 35 days (averaging 23 days), with shorter generation times under high food conditions and low density. We thus conservatively estimate our whole experiment to cover a maximum of 10 overlapping generations, with faster generation times in the beginning of the experiment due to less dense populations. We manipulated food environment to test if a reduction in food availability would exacerbate the hypothesized effects of founder population size on when scramblers are first expressed. We scored when scramblers were first expressed in the populations and assessed if this was related to the long‐term success of populations, measured by how population size, total adult number, population growth, and female fecundity changed over the course of the experiment.

## 2 Materials and Methods

### 2.1 The Bulb Mite

**Figure 1:**
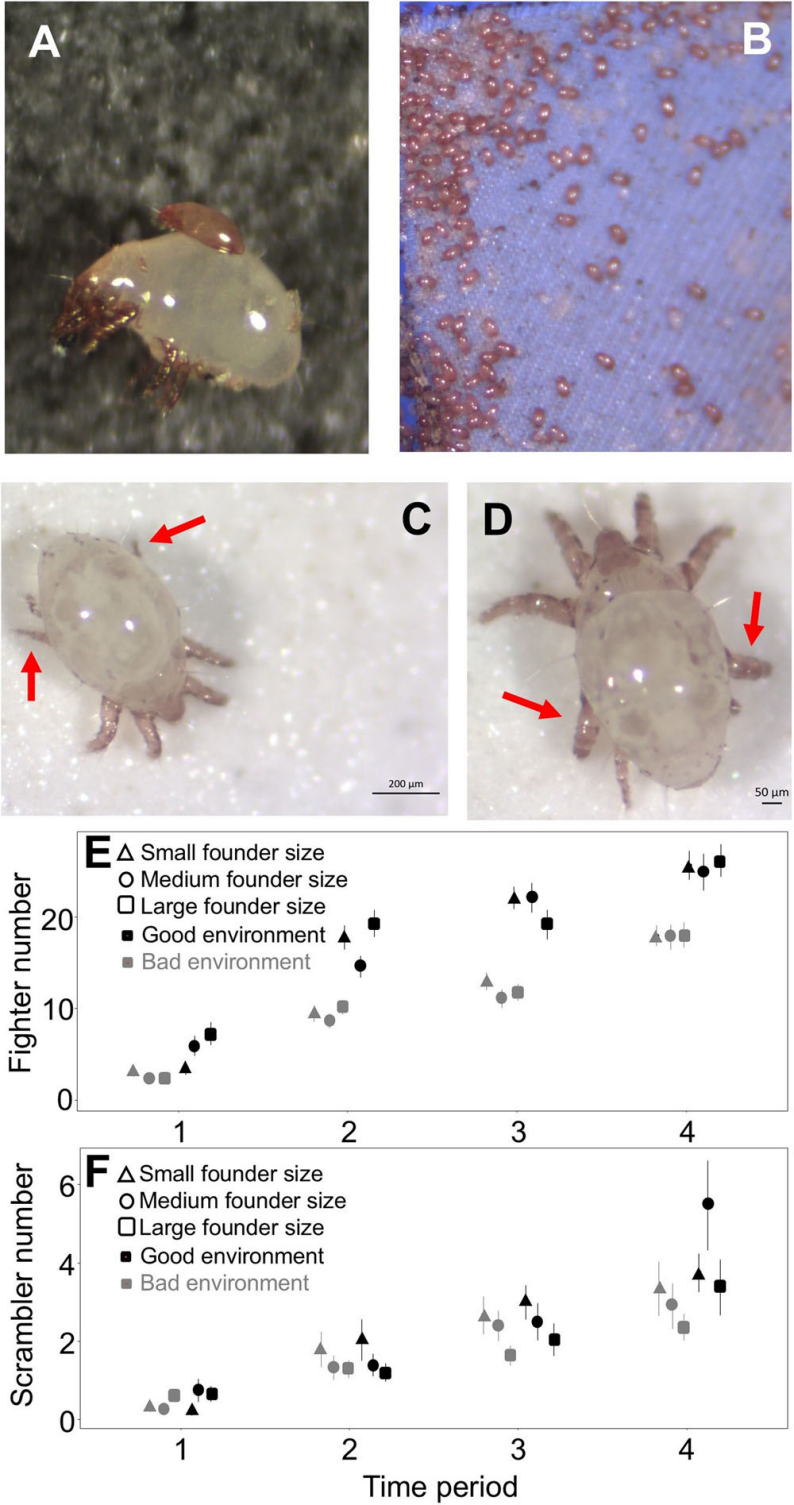
A deutonymph attached to an adult female bulb mite, *Rhizoglyphus robini* (A) and an aggregation of deutonymphs in one of our stock cultures (B). Adult male scrambler (C) and adult male fighter (D) with red arrows indicating muscular, thick fighter legs (D) or normal legs (C). Mean number of fighters ± SE (E) and mean number of scramblers ± SE (F) for all treatment combinations in relation to time periods 1 (Week 3–6), 2 (Week 7–10) 3, (Week 11–14), and 4 (Week 15–18) of the experiment (for some treatment combinations, the symbols cover the error bars). [Color figure can be viewed at wileyonlinelibrary.com]

*R. robini* (Acari: Acaridae), has a short, and environmentally mediated lifecycle: depending on environmental conditions (e.g., temperature and food quality), development to adulthood takes between 11 and 40 days (Smallegange 2011), with sexually reproducing adults living between 31 and 130 days (Gerson et al. 1983; Díaz et al. 2000). Within this developmental transition, 6 distinct stages exist: egg, larvae, protonymph, deutonymph (facultative dispersal stage that only occurs under adverse conditions: Figure 1A,B), tritonymph and adult (Baker 1982), each of which (exception: larval stage) is preceded by a quiescent/sessile molting phase. Only at the adult stage is the bulb mite's sex identifiable; adult males are morphologically and behaviorally dimorphic with fighter males displaying enlarged and thickened third leg pairs with a sharp terminus used to kill conspecifics (Figure 1C), and smaller scramblers that lack the thickened, weaponized third leg pair (Radwan 1995) (Figure 1D). A possible third mating tactic, the megascrambler, may also exist within *R. robini* (Stewart et al. 2018), but because it is so rare, we have not included these individuals within our experimental data.

### 2.2 Specimen Collection and Maintenance

We sourced bulb mites from maintained stock populations originating from 10 sampling sites via flower bulbs near Anna Paulowna, North Holland, Netherlands (2010), that comprise tens‐of‐thousands of individuals. These mites were reared and maintained at the Institute for Biodiversity and Ecosystem Dynamics at the University of Amsterdam, Netherlands, in a controlled incubator (25 ± 1°C, 60% relative humidity, 16:8 h light‐dark photoperiod; *sensu* Smallegange 2011) under a rearing environment commonly used in life history studies to assess growth, development, and adult male phenotype expression of mites from the family Acaridae (e.g., Gerson et al. 1983, 1991). The proportion of adult males that are fighters is > 90% (Smallegange et al. 2018). Mites are given a diet of *ad libitum access* to rolled oats, a nutritional medium that these individuals have adapted to for over a decade.

From these stock populations, we collected 560 deutonymphs over the period of 1 week to initiate populations that represent “founder populations” (see further Section 2.3) within 12 mL tubes (40 × 23 mm) filled two‐thirds with a mix of plaster of Paris and charcoal (enabling contrast between mites and the substrate). The plaster mixture was half saturated with water before population initiation to help maintain humidity for the duration of the experiment, after which one drop of water was added during feeding. Good and bad nutritional environments were enforced by feeding each population either a high (0.5 mg) or low (0.25 mg) amount of oats every 2 days, respectively. Population tubes were covered with punctured caps and mesh (escape prevention) to allow for air ventilation and water evaporation. These populations were then maintained at 25°C with > 70% relative humidity in an unlit incubator.

### 2.3 Experimental Setup

We followed populations for 20 weeks (approximately < 10 overlapping generations, 133 days). Populations were initiated from either small (5 deutonymphs), medium (10 deutonymphs), or large (25 deutonymph) founder sizes and put in a good or bad nutritional environment. All treatment combinations were replicated seven times each and allowed to freely evolve without intervention. Population demographics were visually observed through a Zeiss Stemi 2000‐C microscope and number of eggs, number of individuals at each life stage, adult sex, and adult male phenotype were scored every 7 days with minimal invasiveness.

### 2.4 Statistical Analyses

Logistical restrictions forced an early end to our experiment for half of the populations at 18 weeks, thus we removed Weeks 19 and 20 from further analysis for all populations. Due to overdispersion of our data from population growth initiated by deutonymphs (abundance of zero data points at the beginning of the experiment), the first 2 weeks of data collection (generation 0) were also discarded. Two populations crashed at Week 6 (one small population from a good environment; one small population from a bad environment), both of which were removed from the analyses. In total, 39 populations remained within our data set, evolving across Weeks 3–18.

We first tested for significant differences in when scramblers were first expressed, quantified as (i) the first day of scrambler expression (Julian day since the start of the experiment) divided over total population size, that is, per capita first day of scrambler expression, and (ii) as the absolute day scramblers were first expressed, across the two food environments (***E***; bad, good) and three founder population sizes (***P***; small, medium, large) and their two‐way interaction, using a general linear model (GLM). In two populations, scramblers were never present and these were removed from this analysis (which did not qualitatively affect the results). Next, we tested for any effects of time period (***T***; binned 4‐week intervals 1, 2, 3, 4), environment (***E***; good, bad), and founder population size (***P***; small, medium, large), and all their interactions, on scrambler expression, total population size, total number of adults, weekly population growth (total population size at week *t* divided over total population size at week *t*‐1), and female fecundity (calculated as the total number of eggs offset to the number of adult females) using Generalized Linear Mixed Models (GLMMs) with population tube as a random factor in R (R Core Team 2022). Scrambler expression was assessed as the total number of scramblers, offset against all adult males (scramblers and fighters) within a population; female fecundity was assessed as the total number of eggs, offset against all adult females within a population. We chose to analyze female fecundity and scrambler expression with an offset term and Gaussian distribution (as opposed to using a binomial error distribution) because our data are counts, and the offset term can model these as proportions and account for the different sample sizes in our experiment. The factor time period ***T*** was created by binning time periods into 4‐week intervals (time periods 1–4 across experimental Weeks 2–18) (smaller time intervals produced qualitatively the same results (see Figshare repository: Stewart and Smallegange 2023), and we chose to proceed with 4‐week intervals for interpretive simplicity).

The GLM(M) model assumptions of Gaussian errors and homoscedacity were confirmed by inspecting the probability plots and error structures. All statistical analyses were performed using Rstudio v.4.0.3 (R Core Team 2022). The package “lme4” was used to create and analyze the GLMMs. For the GLMMs, we used model simplification to acquire the best model from the full models (Crawley 2007). For brevity, the least significant term, starting with the highest order interaction, was first removed from the fitted model to produce a reduced model. If this removal led to a significant increase in deviance, we did not remove this term from the model and removed the second least significant term, again starting with the then highest order interaction. If a removal did not lead to a significant increase in deviance, then this term was removed from the model, and the next least significant term of the highest order interaction was removed (*sensu* Van den Beuken et al. 2019a). Deviance significance was tested using a likelihood ratio test. We repeated these steps until only significant terms remained in the model. Datasets generated and analyzed, and R‐code, are available in the Figshare repository (Stewart and Smallegange 2023).

## 3 Results

### 3.1 First Day of Scrambler Expression

**Figure 2:**
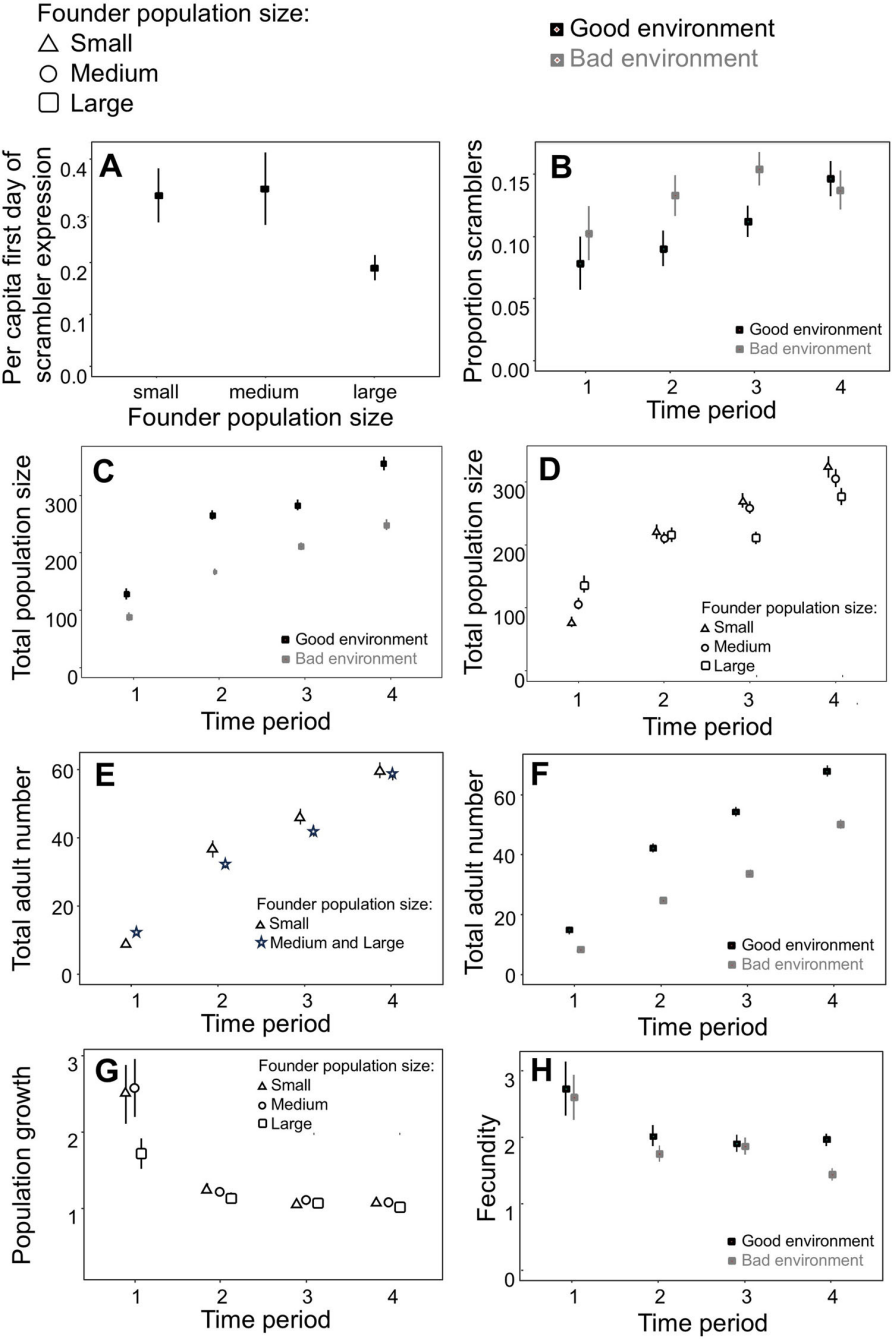
Experimental results. (A) per capita scrambler first day of scrambler expression (the first day scramblers were expressed, divided over total population size) for each founder population size, and the proportion of males that are scramblers (B), total population size (C), and total number of adults (E) for the two food environments (good: black symbols, bad: gray symbols), and total population size (D) and population growth (G) for different founder population size (large: squares, medium: circles, small: triangles) in relation to time periods 1 (Week 3–6), 2 (Week 7–10) 3, (Week 11–14), and 4 (Week 15–18) of the experiment. Adult numbers did not differ over time between the medium and large founder populations (see main text) but both differed from the small founder populations in relation to the different time periods (F, large and medium: starts, small: triangles), as did female fecundity (H) for the two food environments. Vertical lines are standard error bars; in some panels, the symbols cover the error bars.

The mean number of fighters and scramblers increased over the course of the experiment (Figure 1E,F). On average, scramblers were expressed 33.2 ± 13 (SD) days after population colonization (i.e., the start of the experiment). Expressing the day of first scrambler expression as per capita, and removing two outliers following residuals analysis because they violated the assumption of normality and homoscedacity of errors, we found a significant effect of founder population size (***P***: *t* = 2.26, *p* = 0.031) with scramblers being expressed earlier in large than in medium and small populations (Figure 2A). There was no significant effect of environment (***E***: *t* = −0.06, *p* = 0.952), or the interaction (***E *** × *** P***, *t* = −1.30, *p* = 0.204). Defining the day scramblers were first expressed as absolute day of scrambler expression, we found no significant treatment effects (***E *** × *** P***, *t* = −0.91, *p* = 0.370; ***E***: *t* = 0.86, *p* = 0.397; ***P***: *t* = 0.1.34, *p* = 0.189).

### 3.2 Scrambler Expression, Population Size, Total Adult Number, Population Growth and Female Fecundity

Overall scrambler expression, that is, the proportion of all males that are scramblers, significantly increased over the course of the experiment, but quicker when populations were in the bad environment than in the good environment (significant interaction ***E *** × *** T***, $\chi_{2}^{2}$ = 25.51, *p* < 0.001) (Figure 2B). The three‐way interaction ***E *** × *** P *** × *** T*** was nonsignificant ($\chi_{6}^{2}$ = 8.29, *p* = 0.218), and neither was the two‐way interaction ***E *** × *** P*** ($\chi_{2}^{2}$ = 0.25, *p* = 0.882), the two‐way interaction ***P *** × *** T*** ($\chi_{2}^{2}$ = 10.46, *p* = 0.107), and the main effect founder population size ***P*** ($\chi_{2}^{2}$ = 0.857, *p* = 0.652).

Total population size increased over the course of the experiment but to a greater extent in the good environment than in the bad environment (significant interaction ***E *** × *** T***, $\chi_{2}^{2}$ = 20.39, *p* < 0.001) (Figure 2C), and differently for each founder population size (significant interaction ***P *** × *** T***, $\chi_{2}^{2}$ = 45.07, *p* < 0.001) (Figure 2D), with small founder populations starting out at the lowest sizes, but reaching the largest sizes by the end of the experiment. The three‐way interaction ***E *** × *** P *** × *** T*** ($\chi_{6}^{2}$ = 6.97, *p* = 0.323) and the two‐way interaction ***E *** × *** P*** ($\chi_{2}^{2}$ = 1.54, *p* = 0.464) were both nonsignificant.

Like total population size, the total number of adults increased over the course of the experiment but to a greater extent in the good environment than in the bad environment (significant interaction ***E *** × *** T***, $\chi_{2}^{2}$ = 31.99, *p* < 0.001) (Figure 2E), and differently for each founder population size (significant interaction ***P *** × *** T***, $\chi_{2}^{2}$ = 15.89, *p* = 0.014) (Figure 2F), with small founder populations starting out at the lowest sizes, but reaching the largest sizes by the end of the experiment (total adult numbers did not differ between medium‐sized and large populations over time, $\chi_{4}^{2}$ = 6.17, *p* = 0.189 and were therefore lumped [Figure 2F]). The three‐way interaction ***E *** × *** P *** × *** T*** ($\chi_{6}^{2}$ = 10.35, *p* = 0.111) and the two‐way interaction ***E *** × *** P*** ($\chi_{2}^{2}$ = 0.40, *p* = 0.818) were both nonsignificant.

Weekly population growth declined over the course of the experiment, but tended to do so the fastest for the small and medium founder populations: their population growth rate in time period 1 was higher than that of the large founder populations before population growth rate leveled off to comparably low values for all population towards the end of the experiment (borderline, nonsignificant interaction ***P *** × *** T***, $\chi_{6}^{2}$ = 10.93, *p* = 0.091) (Figure 2G). All other effects were nonsignificant (***E *** × *** P *** × *** T***: $\chi_{6}^{2}$ = 2.60, *p* = 0.857; ***E *** × *** P***: $\chi_{2}^{2}$ = 0.70, *p* = 0.704; ***E *** × *** T***: $\chi_{2}^{2}$ = 0.65, *p* = 0.884; ***E***: $\chi_{1}^{2}$ = 0.034, *p* = 0.854).

Female fecundity significantly decreased over the course of the experiment but especially in the bad environment toward the end of the experiment (significant interaction ***E *** × *** T***, $\chi_{2}^{2}$ = 17.78, *p* < 0.001) (Figure 2H). The three‐way interaction ***E *** × *** P *** × *** T*** ($\chi_{6}^{2}$ = 2.54, *p* = 0.864), the two‐way interaction ***E *** × *** P*** ($\chi_{2}^{2}$ = 0.11, *p* = 0.944), the two‐way interaction ***P *** × *** T*** ($\chi_{2}^{2}$ = 5.82, *p* = 0.443), and the main effect founder population size ***P*** ($\chi_{2}^{2}$ = 0.285, *p* = 0.867) did not significantly impact female fecundity.

## 4 Discussion

Whether developmental plasticity makes any difference to phenotype dynamics and adaptive evolution depends primarily on how development responds to environmental change. These responses can alter the phenotype distribution that selection acts upon and not directly on how much heritable variation there is (Uller et al. 2024). In fact, developmental plasticity, particularly for environmentally induced phenotypes like the dispersal and alternative adult male phenotypes in our bulb mite model system, have been shown to demonstrate persistent and adaptive directionality on evolution (West‐Eberhard 2003). In our bulb mite study system, we aimed to differentiate between “generative” or “selective” processes for the evolutionary rescue of populations by hypothesizing that, on the one hand, scrambler expression in small founder populations is a mechanism to rescue populations from impending collapse and alleviate some of the negative fitness consequences of inbreeding depression—our “selective” hypothesis. Our alternative hypothesis was that, all else being equal, small founder populations can favor fighter expression because per capita food availability will be higher allowing more juvenile males to survive and mature when investing in costly fighter morphology—our “generative” hypothesis.

Our findings support the “generative” hypothesis as scramblers were expressed for the first time later in the small founder populations compared to the medium and large founder populations. What is more, we found a trend that these late‐scrambler‐expression populations (small founder populations) showed higher growth at the start of the experiment than early‐scrambler‐expression populations (large founder populations). Thus, even though their population size, including total adult number, was the lowest at the start of the experiment, the small founder populations ultimately achieved the highest population size at the end of the experiment. Seemingly, small founder populations, if able to overcome the occasional extinction events (our study *n* = 2), suffered little if any adverse long‐term effects of colonization size, and consequently performed even better in terms of their ultimate size they could attain compared to large founder populations.

Resource availability is likely central to explaining our population dynamic results, as it is a key dimension of the environment that affects the survival, development, and reproduction of individuals (Ellis et al. 2009). At the start of the experiment, per capita food availability was highest in the small founder populations, possibly explaining their high population growth rate that kept these founder populations at a higher growth trajectory than the other populations. At the same time, the high per capita food availability meant that fewer males suffered (local) food stress (Rhebergen et al. 2022) so that scramblers were first expressed later in the small founder populations. In fact, scrambler expression overall was higher in the bad food environment during most of the experiment. While populations under bad food environments suffered smaller average populations sizes, food environment itself did not impact population growth. In concurrence with founder population sizes, it is possible that the food levels we chose were not different enough to elicit a distinctive population growth response. For example, food environment did not impact female fecundity until the very end of the experiment. What is more, in field populations, colonization of new food environments was not associated with strong population bottlenecks in bulb mites (Przesmycka and Radwan 2023). All this could explain why we did not find that food environment exacerbated either of our hypothesized responses to founder population size. Finally, fighters can cannibalize especially under starvation (Van den Beuken et al. 2019b). Cannibalism can impact population structure and size in polymorphic populations like bulb mites in complex ways (Croll et al. 2019). However, we do not know yet to what extent these effects depend on the food environment within a population, nor do we know if or how this can impact inbreeding depression. Assuming fighters were disproportionately killing scramblers in our founder populations, especially under unfavorable food conditions (i.e., the largest populations), then our finding would be an underestimation of the first‐day scramblers were expressed in the founder populations, as we could have missed already cannibalized scramblers in our counts. Thus, even if cannibalism occurred, the fact that we still find that scramblers were expressed earliest in the densest populations supports the “generative” hypothesis.

Previous studies have found that male deutonymhs matured only into fighters (or females) and not scramblers upon colonization (Smallegange and Coulson 2011; Deere et al. 2015). But in two populations in our study (a medium and large founder population), the first adult males to mature from a deutonymph were scramblers, raising the question what exactly is the relationship between deutonymph and male morph expression, and how does it come about? Deutonymph and fighter expression both require energy (Deere et al. 2015; Rhebergen 2022). For example, juveniles mature at a smaller size if they go through the deutonymph stage than if they do not (Deere et al. 2015), but this smaller body is energetically cheaper to develop, grow and maintain. Thus, if there is sufficient food available, like there was in the aforementioned studies (at ad libitum access to high‐quality food: Smallegange and Coulson 2011; Deere et al. 2015), it is likely that at maturation, these smaller, deutonymph‐derived juvenile males all accumulate sufficient resources for fighter leg development. But, if food availability is low, for example, due to density‐dependent competition over food, as in our experiment, juvenile males are less likely to reach the resource threshold for fighter leg development resulting in a higher propensity of scrambler expression. Our sample size of scramblers maturing from deutonymphs is very low (3 of 560 deutonymphs; 0.005%), but it would be interesting to explore this relationship in more detail in future studies.

The key question we asked was whether scrambler expression within the founder population would allow a population to better survive and proliferate (evolutionary rescue), or whether such an adaptive phenotypic response is mostly due to a developmental plasticity? Whereas the former seeks explanations at the genetic level, assuming a direct link between genotype and phenotype (e.g., Foster 2005; Abdul‐Rahman et al. 2021), the latter places explanations at the phenotype level. The assumption of a direct genotype‐phenotype link can be hard to justify when fundamentally the resources an organism has available must be traded‐off between somatic and reproductive effort (Stearns 1992). This then results in a wide range of life history trajectories that tend not to be genetically fixed but show adaptive developmental plasticity (Pigliucci 2001; West‐Eberhard 2003; De Roos and Persson 2013). Thus, when we seek to understand the developmental causes of adaptive evolution during colonization, we need to understand how resource limitation stress shapes adaptive responses to changing environmental conditions (Ellis and Del Giudice 2019). In bulb mites, scrambler expression is regulated by the dynamics of food competition and individual energy economies (Rhebergen et al. 2022). Because the body size threshold for male morph expression shifts toward lower values under low food quality (Rhebergen et al. 2022), juvenile males are more likely to develop into a scrambler in large founder populations because in such scenarios, density‐dependent food competition is relatively high. Thus, if juvenile males alleviated food stress in the large founder populations by maturing into a scrambler and then survive (instead of investing into costly fighters but then die during metamorphosis), this developmental response can adaptively mitigate the negative consequences of adverse conditions (Nettle and Bateson 2015; Smallegange et al. 2019). Developmental trajectories could then similarly rescue populations from collapse. It can also explain why experimental bulb mite populations selected for fighter expression are more prone to extinction under environmental change (Łukasiewicz et al. 2023), as in those populations, more males are on a fighter development trajectory but die during maturation.

Adaptive, mitigating developmental plasticity is likely to have evolved from nonadaptive plasticity as a by‐product of the physics and chemistry of development that evolve to produce adaptive somatic mechanisms that mitigate immediate stress (Nijhout 2003). Over evolutionary time, these adaptive somatic stress‐coping mechanisms can be evolutionarily liable to be triggered by additional, anticipatory mechanisms that produce adaptive results (anticipatory developmental plasticity) (Smallegange et al. 2019). If true, this shows how development can be a complex cause of phenotypic variation that biologists need to satisfactorily explain both short‐ and long‐term evolutionary patterns (Smallegange 2022; Deere and Smallegange 2023).

## Author Contributions

This study was designed by Kathryn A. Stewart. Kathryn A. Stewart and Isabel M. Smallegange performed the analyses and co‐wrote the manuscript.

## Data Availability

Datasets generated and analyzed, and R‐code, are available in the Figshare repository (Stewart and Smallegange 2023). The data that support the findings of this study are openly available in Figshare at https://doi.org/10.6084/m9.figshare.22082219.v4, reference number v4.
